# Calculating temperature-dependent X-ray structure factors of α-quartz with an extensible Python 3 package

**DOI:** 10.1107/S1600576722005945

**Published:** 2022-07-28

**Authors:** John P. Sutter, James Pittard, Jacob Filik, Alfred Q. R. Baron

**Affiliations:** a Diamond Light Source Ltd, Harwell Science and Innovation Campus, Chilton, Didcot, OX11 0DE, United Kingdom; b School of Physics, HH Wills Physics Laboratory, University of Bristol, Tyndall Avenue, Bristol, BS8 1TL, United Kingdom; c Materials Dynamics Laboratory, RIKEN SPring-8 Center, 1-1-1 Kouto, Sayo, Hyogo 679-5148, Japan; SLAC National Accelerator Laboratory, Menlo Park, USA

**Keywords:** quartz, X-rays, structure factors, Python

## Abstract

The design of X-ray optics based on diffraction from crystals depends on the accurate calculation of the structure factors of their Bragg reflections over a wide range of temperatures. In general, the temperature dependence of the lattice parameters, the atomic positions and the atomic thermal vibrations is both anisotropic and nonlinear. Implemented here is a software package for precise and flexible calculation of structure factors for dynamical diffraction. α-Quartz is used as an example because it presents the challenges mentioned above and because it is being considered for use in high-resolution X-ray spectroscopy. The package is designed to be extended easily to other crystals by adding new material files, which are kept separate from the package’s stable core. Python 3 was chosen as the language to allow the easy integration of this code into existing packages. The importance of a correct anisotropic treatment of the atomic thermal vibrations is demonstrated by comparison with an isotropic Debye model. Discrepancies between the two models can be as much as 5% for strong reflections and considerably larger (even to the level of 100%) for weak reflections. A script for finding Bragg reflections that backscatter X-rays of a given energy within a given temperature range is demonstrated. The package and example scripts are available on request. Also discussed, in detail, are the various conventions related to the proper description of chiral quartz.

## Abstract 2

A Python 3 software package for precise calculation of X-ray structure factors of α-quartz over a wide temperature range is presented. α-Quartz was chosen because of its practical application in high-resolution X-ray spectroscopy, but this software package can be easily extended to other crystals.

## 1. Introduction

For decades, materials scientists, chemists and biologists have obtained valuable and detailed information on the electronic structure and phonon spectra of numerous materials by applying different types of inelastic X-ray scattering, including resonant inelastic X-ray scattering (RIXS) (Schülke, 2007 ▸; Ament *et al.*, 2011 ▸), X-ray nuclear resonant scattering (Gerdau & de Waard, 1999 ▸, 2000 ▸) and millielectronvolt-resolution non-resonant inelastic X-ray scattering (Baron, 2016 ▸). Different approaches have been used to make the optics for these experiments. For hard X-rays (>5 keV), optics typically employ Bragg reflections in perfect crystals, as these have sufficient energy resolution for inelastic measurements. However, the acceptance of Bragg reflections in flat perfect crystals is typically ∼1–100 µrad, which limits the solid angle of scattered radiation that can be collected from a sample. Thus, to improve rates, most inelastic scattering measurements employ figured optics to increase the analyser acceptance. For experiments with roughly electronvolt resolution, crystal wafers can be directly bent into the desired shape (often either cylindrical or spherical) while, for better resolution, some sort of dicing is done, with many perfect crystallites attached to a figured substrate – the dicing avoids degradation of the resolution due to the strain caused by bending. However, more recently, it has also become possible to use a combination of a collimating optic with a flat crystal (*e.g.* Shvyd’ko *et al.*, 2014 ▸), also called a ‘post-sample collimation’ or ‘PSC’ geometry (Baron, 2016 ▸). This potentially offers advantages, especially for high resolution, as one can avoid the difficulty of figuring an optic without distorting it unacceptably.

It is useful to have access to different perfect crystal materials. While the various geometries, including figured analysers or post-sample collimation, improve the angular acceptance, the efficiency for a given energy resolution is improved if one can work near Bragg angles of 90°, that is ‘backscattering’. The crystal must then diffract from a set of atomic planes whose spacing *d* is half the wavelength used in the experiment, with a usually small range of tunability before losses or spectral broadening become an issue. Therefore, it is very convenient to have computational tools that deal easily with different, and potentially complex, crystal types. In particular, the high symmetry of the cubic diamond structure’s space group 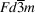 , in which silicon, the most common material for X-ray optics, also crystallizes, results in a rather limited number of possible *d* spacings and thus a restricted choice among only a few energies. The high symmetry also increases the chance that unwanted Bragg reflections can be simultaneously excited near backscattering and interfere with the backscattering reflection (Sutter *et al.*, 2001 ▸; Huang *et al.*, 2014 ▸). These parasitic reflections (also sometimes called ‘*umweg*’ or ‘multibeam’ conditions) can be very numerous: the 12 4 0 reflection, which reaches backscattering at 14 438 eV, close to the Mössbauer resonance of the ^57^Fe nucleus, has 22 of them. Germanium and diamond crystallize in the same space group and therefore suffer from the same weaknesses as silicon.

Materials with lower-symmetry structures are considered for use in X-ray spectrometers, as they offer a better chance of having a backscattering Bragg reflection close to a desired energy. These materials must be available in large highly perfect ingots so that variations in lattice-plane spacing do not broaden the energy resolution. They must be able to be cut, polished and etched, to bend elastically without fracture, and to withstand intense radiation. Yavaş *et al.* (2017 ▸) have reviewed some options, which all form trigonal crystal structures: α-quartz (SiO_2_), lithium niobate (LiNbO_3_) and sapphire (Al_2_O_3_). Of these three, α-quartz offers the best crystal quality, as well as the best choice of Bragg reflections when an energy resolution below 60 meV for X-rays of 5–10 keV energy is desired. α-Quartz wafers and ingots grown synthetically by the hydrothermal process (Brice, 1985 ▸; Laudise, 1987 ▸) are commercially available. Sutter *et al.* (2005 ▸, 2006 ▸) were the first to demonstrate their high quality, with 4 meV resolution attained over 11 cm^2^ at 10 keV (Δ*E*/*E* = 4 × 10^−7^), and Imai *et al.* (2007 ▸) quickly added further confirmation. Hönnicke *et al.* (2013 ▸) have shown similar quality (Δ*d*/*d* = 5 × 10^−7^) over an area of 79 × 32 mm. α-Quartz crystals can therefore be a useful addition to the X-ray optics developer’s toolbox.

The development of both bent-crystal and flat-crystal X-ray spectrometers that use α-quartz crystals has been actively pursued during the past decade. Honnicke *et al.* (2016 ▸) have constructed a diced quartz spherical analyser and used it to examine the Ni *K*α emission spectrum around 7.47 keV. Their analyser was curved to a radius of 1 m and had 360 crystal blocks of area 1.5 × 1.5 mm, yielding a total collection area of 810 mm^2^. Said *et al.* (2018 ▸) used a similar quartz analyser to perform RIXS on the 11.215 keV Ir *L*_3_ emission line with an energy resolution of 10.53 meV. Their analyser was bent to a 2 m radius and was made from a quartz wafer of 25 mm diameter, yielding a total area of 490 mm^2^. Kim *et al.* (2018 ▸) improved the resolution at the Ir *L*_3_ emission line to an estimated 3.9 meV by using a flat α-quartz crystal analyser with post-sample collimation; Pereira *et al.* (2015 ▸) spherically bent an α-quartz crystal of ∼0.1 mm thickness and 40 × 60 mm cross section down to a radius of 672 mm. We note that α-quartz is more susceptible than silicon to distortion under thermal loads (having both lower thermal conductivity and higher thermal expansion), as has been demonstrated for flat-crystal high-resolution monochromators (Gog *et al.*, 2018 ▸), but the analyser crystals in X-ray spectrometers generally have small or negligible thermal loads. Moreover, the large thermal expansion of quartz makes it tunable in energy over wider ranges than silicon can reach for the same temperature change. Quartz is thus an important material for such optics.

The performance of an X-ray optic, particularly the bandpass and the efficiency, depends sensitively on the structure factor of the crystal’s Bragg reflection. When using backscattering Bragg reflections, it is also necessary to take the temperature of the crystal into account, because the temperature changes the interplanar spacing *d* and hence the selected X-ray energy. Therefore, accurate calculation of the structure factor for a wide range of Bragg reflections, X-ray energies and crystal temperatures is necessary for the optimal design of an X-ray spectrometer. This task is more complex for α-quartz than for silicon because the positions of the atoms of α-quartz within the unit cell depend on temperature, unlike those of silicon, and because the thermal vibrations of the atoms in α-quartz are strongly anisotropic. Moreover, α-quartz has a chiral structure, being able to crystallize in either left-handed or right-handed forms that are mirror images of each other. The variety of methods used in the literature to describe the crystal structures of α-quartz has led to confusion that persists to this day (Huang *et al.*, 2018 ▸; Glazer, 2018 ▸). A practical algorithm for calculating the structure factors of α-quartz not only must be able to accommodate this diversity of conventions but also should serve as a template for the rapid calculation of large numbers of structure factors of Bragg reflections from even more complex crystals.

We have written a new software package designed to calculate the structure factors of Bragg reflections in α-quartz at temperatures from 20 to 838 K while giving each user the freedom to choose the description of the crystal structure. The language used is Python 3, which permits both stand-alone operation and easy integration into widely used software packages such as *OASYS* (Sánchez del Río & Rebuffi, 2019 ▸) and *SRW* (Chubar & Elleaume, 1998 ▸). The software is constructed so that all information about the diffracting crystal is contained in a material file separate from the code that calculates the structure factor. Users may write a different material file for any convention of α-quartz they wish. They may also write separate material files for different models of the thermal vibration in α-quartz (*e.g.* anisotropic displacement ellipsoids versus Debye temperature). Two examples will demonstrate the strong effect of the temperature dependence of the atomic positions and thermal vibrations on the structure factors, particularly on those of weak reflections. Even for strong reflections, a full anisotropic treatment of the atomic Debye–Waller factors can yield structure factors with magnitudes differing by as much as 5% from those calculated with an isotropic Debye model. A script for finding Bragg reflections that reach backscattering at a given energy within a given range of temperatures while providing suitable efficiency and bandpass is also provided. This software package can be extended to the calculation of structure factors of any other crystal simply by writing an appropriate material file. As a result, this software package is named *PyCSFex* (pronounced ‘pixfex’) for ‘Python crystal structure factor extensible’.

## 2. Crystal structure of α-quartz

α-Quartz, also called low quartz, is one of the many forms that SiO_2_ can assume under various temperatures and pressures (Frondel, 1962 ▸; Johnson & Foise, 1996 ▸), and it is the only crystalline form that is thermodynamically stable at room temperature and atmospheric pressure (although glass and some other crystalline forms are metastable under these conditions). At atmospheric pressure, SiO_2_ exists stably as α-quartz up to 846 K, at which it undergoes a phase transition to (high) β-quartz. On the way from there up to its melting point of 1996 K, SiO_2_ passes through several other crystalline phases (Brice, 1985 ▸).

Disagreements in the description of the α-quartz crystal structure involve conflicting definitions of the crystal’s handedness, the choice of using right-handed or left-handed coordinate axes to describe the atomic positions, two different ways of labelling the diffracting planes, and finally the choice of coordinate origin. Donnay & Le Page (1978 ▸) reviewed the various options and gave the atomic positions in each. Their review may help improve currently available software packages such as *XOP* (Sánchez del Río & Dejus, 2004 ▸, 2011 ▸) and the APS *X-ray Server* (Stepanov, 1997 ▸). Gross errors in the structure factor calculation can occur if the software’s conventions are not the ones that its users have in mind, as will be seen below. In the following section, we will summarize the various conventions that have been used to describe the crystal structure of α-quartz. We will explain our preferences but, as we wish to be inclusive, we will also explain how those who prefer other conventions can convert ours into theirs with minimal effort.

Crystals of α-quartz form a trigonal Bravais lattice in either of two space groups, *P*3_1_21 and *P*3_2_21, that are mirror images (enantiomorphs) of each other. They are composed of SiO_4_ tetrahedra that are linked together at their corners and are slightly distorted so that one long type (1.6145 Å) and one short type (1.6101 Å) of Si—O bond are present (Nuttall & Weil, 1981 ▸). In this paper as in most others, a hexagonal unit cell with three SiO_2_ formula units is used. The lattice coordinate vectors **a** and **b** are of equal length *a* and form an angle of 120°, and the lattice coordinate vector **c** has length *c* and is perpendicular to both **a** and **b**. The *c* axis is a threefold (not sixfold) screw axis, which may be left-handed or right-handed according to the following definition:

(i) Wrap the fingers of the right hand around the *c* axis while extending the right thumb along the positive *c* direction.

(ii) Rotate the crystal structure by 120° in the direction in which the fingers of the right hand curl.

(iii) If a translation of the rotated crystal structure by **c**/3 brings the crystal structure back to its original state, the screw axis is right-handed.

(iv) If a translation of the rotated crystal structure by −**c**/3 brings the crystal structure back to its original state, the screw axis is left-handed.

We repeat this definition to resolve the confusion that can be caused by the existence of both a slightly distorted sixfold helix of Si atoms and an exact threefold helix of Si atoms that have opposite handedness (Glazer, 2018 ▸). Application of these rules to either of these helices will yield the same result for the handedness of the screw axis. Perpendicular to the screw axis, there are three symmetrically related twofold axes that are separated in angle by 120° and in height by *c*/3. One Si atom lies on each of these three axes. The three Si atoms and six O atoms of the hexagonal unit cell are at Wyckoff positions (*a*) and (*c*), respectively.

Part of the confusion in the literature is that different communities define a chiral crystal’s handedness in ways that for α-quartz (though not for all chiral crystals) are directly opposite.^1^ In this paper, we will therefore avoid the use of the terms ‘left-handed quartz’ and ‘right-handed quartz,’ which though simple are ambiguous. Instead, we will use the Latin terms ‘dextro’ and ‘laevo,’ which are common in the literature and are defined as follows (Donnay & Le Page, 1978 ▸):

*Dextro*: rotates the plane of polarization clockwise as seen by an observer looking upstream. The screw axis *c* is left handed. The space group as given by *International Tables for Crystallography* (2016 ▸) is No. 154, *P*3_2_21.

*Laevo*: rotates the plane of polarization counterclockwise as seen by an observer looking upstream. The screw axis *c* is right handed. The space group as given by *International Tables for Crystallography* (2016 ▸) is No. 152, *P*3_1_21.

The best choice of handedness for the coordinate axes has also been much discussed over the years. Donnay & Le Page (1978 ▸) cited numerous studies predating 1930 in which a left-handed coordinate system was used, but they stated that since then the right-handed coordinate system had become more common. However, they did support the idea of matching the handedness of the coordinates to that of the screw: that is, using a left-handed coordinate system for dextro quartz and a right-handed coordinate system for laevo quartz. α-Quartz crystals often appear in nature as mirror-imaged (Brazil) twins, and this proposal would permit consistent Miller indexing across both members of such a twin. This proposal would also allow the atomic positions in both dextro quartz and laevo quartz to be assigned the same set of values. According to Glazer (2018 ▸), crystal growers have in fact adopted this idea, but in the opposite sense, using a right-handed coordinate system for dextro quartz and a left-handed coordinate system for laevo quartz. However, Glazer himself uses a right-handed coordinate system for both dextro and laevo quartz, and we ourselves are accustomed to using right-handed coordinate systems in our daily work; therefore, we have chosen to use right-handed coordinate systems regardless of the quartz crystal’s handedness.

Even after these choices have been made, the directions of the lattice vectors **a** and **b** remain to be determined. From the beginning, these axes have been chosen to be parallel to two of the three symmetrically equivalent twofold axes on which the Si atoms are located, but this still does not specify which way the positive *a* axis points. A crystal of α-quartz forms three main types of flat faces with distinctly different appearances, defined below. We will use the four-index Miller–Bravais indices (*hkil*), where *i* = −(*h* + *k*), to describe each set of atomic planes in the conventional hexagonal coordinate system. The third index is of course redundant but brings out the crystal symmetries more clearly.

(i) **r** faces: ‘major rhombohedra’, large and often shiny.

(ii) **z** faces: ‘minor rhombohedra’, smaller and duller than **r** faces.

(iii) **m** faces: ‘prismatic’, Miller–Bravais indices 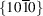 and 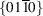 .

Two settings exist for the assignment of Miller–Bravais indices to the atomic planes:

(i) *r* (obverse) setting: **r** faces are 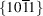 and **z** faces are 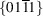 . According to Glazer (2018 ▸), the 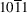 reflection is stronger than the 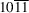 reflection, and the 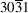 reflection is much stronger than the 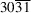 reflection.

(ii) *z* (reverse) setting: **r** faces are 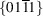 and **z** faces are 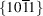 . The 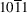 reflection is weaker than the 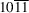 reflection, and the 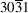 reflection is much weaker than the 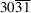 reflection.

The two settings differ by a rotation of the *a* and *b* axes by 180° about the *c* axis.

Though Glazer (2018 ▸) views the obverse setting as the standard, and Donnay & Le Page (1978 ▸) cited several previous authors who used it, the reverse setting is widely used in the literature. Therefore, we have chosen the reverse setting for our program code. Fortunately, material files written for the reverse setting can be easily switched to the obverse setting, as will be shown below.

With a right-handed coordinate system and the reverse setting, laevo quartz is described by the *z*(−) setting and dextro quartz by the *z*(+) setting of Donnay & Le Page (1978 ▸). The plus and minus signs are those of the charge that develops on the positive end of the *a* axis when the crystal is stretched along that axis.

Finally, the literature contains multiple choices of the origin of the coordinate system, which can be classified by the height of the twofold axis that is parallel to the coordinate vector **a**. Many authors, including Donnay & Le Page (1978 ▸) and Glazer (2018 ▸), place the origin so that this height is zero; thus, the first Si atom is located at coordinates (*u*, 0, 0) in both dextro and laevo quartz. However, *International Tables for Crystallography* (2016 ▸) sets this Si atom at 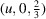 in dextro quartz and 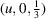 in laevo quartz. This is also the convention followed by Parthé & Gelato (1984 ▸) in their standardized notation. The decisive factor here is that, as pointed out by Glazer (2018 ▸), dextro α-quartz of space group *P*3_2_21 changes above the transition temperature of 846 K into left-screw β-quartz of space group *P*6_2_22. This is No. 180 in *International Tables for Crystallography* (2016 ▸), in which the Si atoms are at Wyckoff position (*c*) and the first Si atom is at 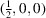 . Likewise, laevo α-quartz of space group *P*3_1_21 changes above the transition temperature into right-screw β-quartz of space group *P*6_4_22. This is No. 181 in *International Tables for Crystallography* (2016 ▸), where again the first Si atom is at 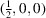 . We follow Glazer (2018 ▸) in using the convention of Donnay & Le Page (1978 ▸) in order to maintain the continuity of the atomic positions across the transition from α-quartz to β-quartz.

Having now passed through the controversies surrounding the α-quartz structure, we conclude this section with a few uncontroversial statements. First, the interplanar spacing *d* of a set of planes with Miller–Bravais indices (*hkil*) in a hexagonal coordinate system is

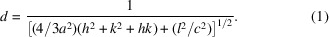




Second, we consider sets of symmetrically equivalent planes. In silicon and germanium, the large number of symmetry operations permits the Miller indices to be permuted arbitrarily and changed individually in sign without altering the diffraction properties. A set of symmetrically equivalent planes {*hkl*} in these cubic materials therefore contains 48 planes (unless two or more of the Miller indices are equal). In α-quartz, on the other hand, a general set of symmetrically equivalent planes {*hkil*} contains at most only six planes, as pointed out by Huang *et al.* (2018 ▸). These are listed in Table 1 ▸.

Note that planes with the same spacing *d* need not always be symmetrically equivalent in α-quartz and therefore need not have the same structure factor. For example, {*hkil*} is not equivalent to {*khil*} if *h* ≠ *k*, and (*hkil*) is not equivalent to 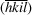 if *h*, *k* ≠ 0. The latter leads to violations of Friedel’s law in cases where the form factors of the atoms have large imaginary parts due to anomalous dispersion. Huang *et al.* (2018 ▸) listed many such cases in their supplementary information.

Finally, because of the low symmetry of the trigonal crystal, very few planes of α-quartz yield forbidden reflections. In *International Tables for Crystallography* (2016 ▸), it is shown that the only forbidden reflections are 000*l* if *l* ≠ 3*n*, where *n* is a non-zero integer.

## 3. Thermal treatment and Debye–Waller factors of α-quartz

For silicon and germanium, the amplitude of the thermal vibrations of the atoms is usually calculated under the assumption of an isotropic Debye model with Debye temperatures of 543 and 290 K, respectively (Batterman & Chipman, 1962 ▸). A similar model has been tried for α-quartz, but different sources give widely different values for the Debye temperature. Gray (1957 ▸) quotes a value of 470 K. Berreman & Chang (1959 ▸) obtained similar values that were dependent on direction: 508 ± 16 K along the *c* axis and 452 ± 15 K perpendicular to the *c* axis. On the other hand, using the measurements of Le Page *et al.* (1980 ▸), Huang *et al.* (2018 ▸) calculated entirely different Debye temperatures of 790.03 K for the Si atoms and 749.31 K for the O atoms. A close look at the original measurements, however, shows that the thermal vibrations are in fact strongly anisotropic. They also show that the Debye model provides a poor fit to the measured mean-square atomic displacements, even when different Debye temperatures are permitted for vibrations along different directions.

The strong anisotropy of the thermal vibrations of the atoms in α-quartz cannot be ignored if the Debye–Waller factors of the Bragg reflections are to be correctly calculated. One must use the full displacement ellipsoids for this purpose. Downs (2000 ▸) has provided a helpful review of this topic. The dimensions and orientation of the displacement ellipsoid of an atom are given in terms of the ellipsoid’s symmetric 3 × 3 matrix β such that the atom’s Debye–Waller factor *D* for a Bragg reflection *hkl* is given by

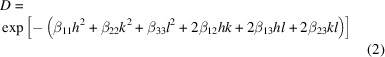

and the mean-square displacement 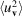 of the atom along some specified vector **v** = *v*_1_ **a** + *v*_2_ **b** + *v*_3_ **c** in real space is given by

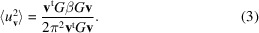

The superscript t denotes the transpose and *G* is the metric matrix, which is also symmetric:

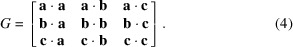




If the atomic thermal motion is isotropic, 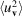 is the same for all real-space vectors **v**. This is fulfilled if β = σ*G* ^−1^, for which 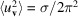 .

## 4. Definitions and values

In Tables 2, 3, 6 and 7, values at 298 K are provided from Kihara (1990 ▸) as examples. These measurements were made on natural clear α-quartz samples. The lattice parameters of other natural and synthetic α-quartz crystals in the literature fall either within or very nearly within the experimental error of Kihara’s measurements (Brice, 1985 ▸; Kihara, 1990 ▸). This good agreement gives confidence that the data presented here are generally applicable. The lattice coordinate system is right-handed and hexagonal. Table 2 ▸ shows the lattice parameters and Table 3 ▸ shows the atomic positions in the lattice coordinate system. The prototype Si atom is located at coordinates (*u*, 0, 0) and the prototype O atom is located at coordinates (*x*, *y*, *z*).

The lattice coordinates of the other Si and O atoms can be generated from those of the prototype atoms by the application of the following symmetry operations:

Dextro: ‘left screw about **c**’. This is a right-handed 120° rotation about **c** followed by a translation of −**c**/3. It transforms a point (*x*, *y*, *z*) into another point (*x*′, *y*′, *z*′) given by

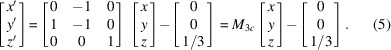




Laevo: ‘right screw about **c**’. This is a right-handed 120° rotation about **c** followed by a translation of **c**/3. It transforms a point (*x*, *y*, *z*) into another point (*x*′, *y*′, *z*′) given by

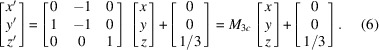




Dextro and laevo: ‘180° rotation about **a**’. This transforms a point (*x*, *y*, *z*) into another point (*x*′, *y*′, *z*′) given by

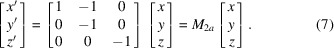




For dextro α-quartz in the *z*(+) setting, the atomic positions and the sequence of symmetry operations by which each atom’s coordinates are generated from those of the prototypes Si1 and O1 are shown in Table 4 ▸, while Table 5 ▸ shows the same information for laevo α-quartz in the *z*(−) setting. In both tables, the rotation matrix *S*, which is the product of all rotation matrices required to obtain each atom’s coordinates from those of its prototype, is provided for the treatment of the displacement ellipsoids and hence the Debye–Waller factor.

The elements of the β matrices of the displacement ellipsoids of the prototype Si atom and the prototype O atom are shown in Tables 6 ▸ and 7 ▸, respectively. Because each Si atom is located on a twofold axis, only β_11_, β_22_, β_33_ and β_23_ are shown for the silicon prototype. The twofold symmetry imposes the relations β_12_ = β_22_/2 and β_13_ = β_23_/2. The elements of the β matrix of the prototype O atom are all independent except that this matrix is symmetric. These values are the same for both dextro α-quartz in the *z*(+) setting and laevo α-quartz in the *z*(−) setting.

To calculate the displacement ellipsoid matrix β′ of another atom from the displacement ellipsoid matrix of its prototype, one uses the equation

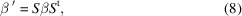

where the value of the rotation matrix *S* for each atom is given in Table 4 ▸ for dextro α-quartz and Table 5 ▸ for laevo α-quartz.

## 5. Conversions between obverse and reverse settings

If a right-handed lattice coordinate system is used, dextro α-quartz can be described in either the reverse *z*(+) setting or the obverse *r*(−) setting of Donnay & Le Page (1978 ▸). Similarly, laevo α-quartz can be described in either the reverse *z*(−) setting or the obverse *r*(+) setting. The switch between reverse and obverse settings is carried out by a 180° rotation of the lattice vectors **a** and **b** about the axis **c**. The parameters listed above must then be converted as in Table 8 ▸.

The importance of sticking to one particular setting when running design calculations for an α-quartz backscattering analyser should be clear from this discussion, since (*hkil*)_obverse_ is not equivalent to 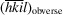 , nor is (*hkil*)_reverse_ equivalent to 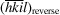 , even though these reflecting planes have the same spacing *d*.

As long as one is consistent in the choice of setting, the structure factor of a reflection *hkil* from dextro α-quartz is equal to the structure factor of the reflection 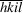 from laevo α-quartz:

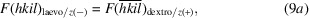




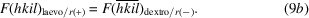




## 6. Temperature dependence of the α-quartz structure

X-ray crystallographic data showing the temperature dependence of the lattice parameters, atomic positions and displacement ellipsoids of α-quartz are scattered across many papers. Table 9 ▸ shows the references selected for this article.

Errors are not clearly given in the data of Barron *et al.* (1982 ▸) but have been estimated here as ±0.0005 Å, since the errors given by Kihara (1990 ▸) for high-temperature lattice parameters are at least this large.

All these experimentally measured temperature dependencies show a common pattern over the temperature range from 13 to 838 K:

(i) A low-temperature region of very small variation with temperature.

(ii) A mid-temperature region of approximately linear variation with temperature.

(iii) A high-temperature region in which the rate of variation with temperature increases rapidly up to the α-quartz → β-quartz transition.

It is desirable to fit the data in all these regions to a single function so that the lattice parameters, atomic positions and displacement ellipsoids can be accurately interpolated from the published data points to any required temperature. However, polynomials of fourth order or below, although they can provide good fits within limited temperature ranges, fail to fit the data well over the full range. Polynomials of fifth order or higher have unphysical oscillations between the data points. An exponential–logarithmic fit function, inspired by the idea of critical exponents, ultimately provided much better fits to the full range of measured data:

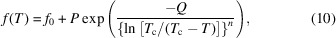

where *T* is the absolute temperature in kelvin and *T*_c_ = 846 K is the transition temperature from α-quartz to β-quartz. *f*(*T*) is the quantity to be fitted (lattice parameters *a* and *c*, Si atomic position *u*, O atomic position *x*, *y*, *z*, displacement ellipsoid β matrix components). The free parameters are *f*_0_, *P*, *Q* and *n*. The conditions *Q* > 0 and *n* > 0 are imposed. Note that the limits of this function are finite:

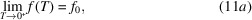




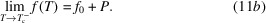




Although this fitting function requires the use of a nonlinear curve-fitting algorithm to determine the optimal parameters, the least-squares Levenberg–Marquardt algorithm (Levenberg, 1944 ▸; Marquardt, 1963 ▸) brings it to a rapid convergence with the measured data. The calculation of the best-fit free parameters was performed using the commercially available software program *OriginPro* (OriginLab, 2017 ▸).

The O-atom position *x* varies too little for a good fit to be made on it alone. More stable fits are achieved by fitting the dependence of the atomic position of the O atom with temperature to a line as follows. The values of *x*, *y*, *z* are converted to Cartesian coordinates using Kihara’s values *a* = 4.9137 Å and *c* = 5.4047 Å at 298 K. The best-fit line to the measured positions of the O atom passes through their centroid **C**. The unit direction vector 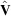 of the best-fit line to the measured positions is determined using standard linear algebra. The equation of the line which gives the best-fit value **r**(*T*) of the O atom’s position in Cartesian coordinates is therefore

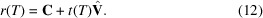

The length *t*(*T*) from the centroid to the point on the best-fit line that is nearest to the O atom’s position at temperature *T* is calculated. The values of *t*(*T*) are fitted using equation (10) to generate an interpolating function that can be evaluated at any temperature within the chosen range. The resulting Cartesian coordinates of the O atom at any temperature are then converted back into the hexagonal coordinates of the α-quartz unit cell. It will be shown that this procedure yields good fits to the experimental values of *x*, *y* and *z*.

The optimal values of the fitting parameters for the lattice parameters, atomic positions and displacement ellipsoids are shown in Table 10 ▸ for dextro α-quartz in the *z*(+) setting. For laevo α-quartz in the *z*(−) setting, it is only necessary to change the signs of *u* for the Si atoms and of *x*, *y* and *z* for the O atoms. As mentioned before, a right-handed hexagonal lattice coordinate system is being used for both dextro and laevo α-quartz.

Note that the zero-temperature values obtained from the best fit to the measured lattice parameters agree well with those estimated by Barron *et al.* (1982 ▸): *a*_0_ = 4.9006 ± 0.0005 Å and *c*_0_ = 5.3979 ± 0.0005 Å. The values measured for the lattice parameters by Lager *et al.* (1982 ▸) have been compared with those determined from the best fit. Their relative deviations from the best fit are ≤2.1 × 10^−4^ for *a* and ≤3.1 × 10^−4^ for *c*.

The best-fit line along which the prototype O atom moves as the temperature varies is

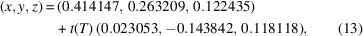

where the best-fit parameters for *t*(*T*) are provided in Table 10 ▸.

**Figure 1:**
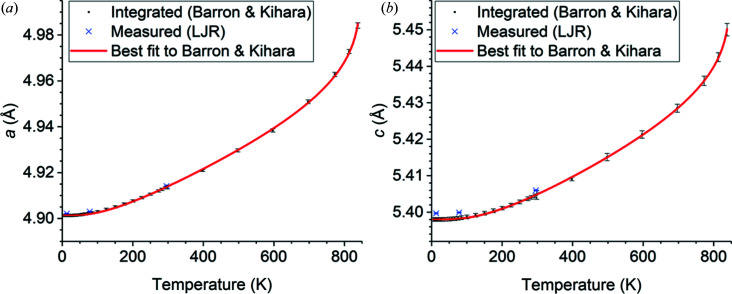
Temperature fits of the lattice parameters (*a*) *a* and (*b*) *c* of α-quartz over the temperature range 13–838 K.

**Figure 2:**
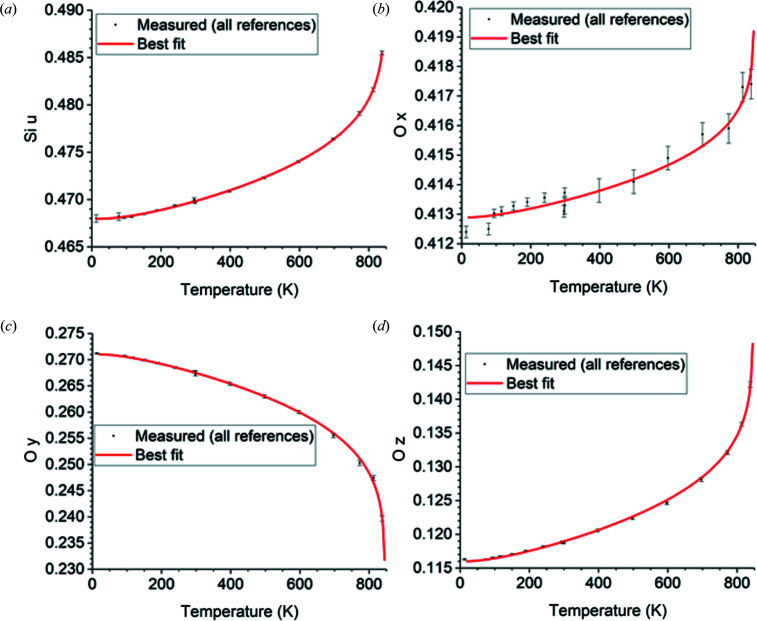
Temperature fits over 13–838 K of the prototype atomic positions (*a*) Si *u* and (*b*) O *x*, (*c*) O *y* and (*d*) O *z* of dextro α-quartz in the *z*(+) setting. For laevo α-quartz in the *z*(−) setting, switch the signs of all these values. See Table 8 ▸ for conversion to the obverse settings *r*(−) for dextro α-quartz and *r*(+) for laevo α-quartz.

**Figure 3:**
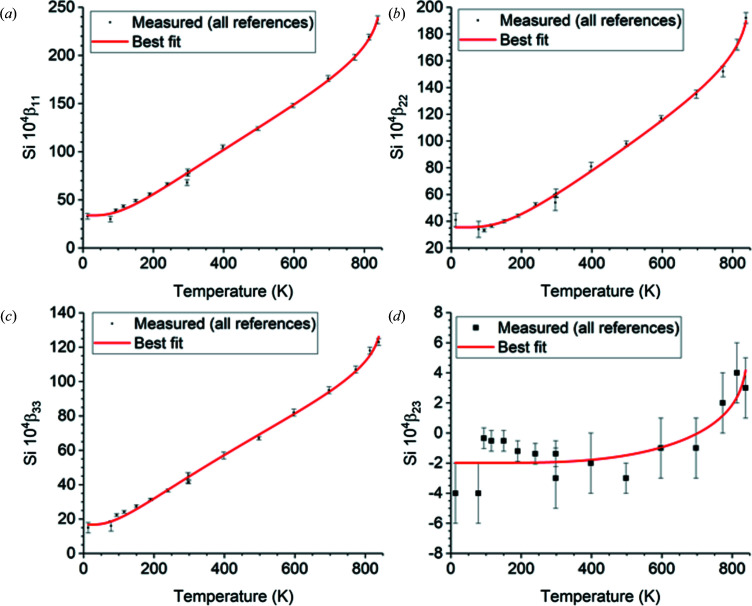
Temperature fits over 13–838 K of the independent displacement ellipsoid matrix elements of the prototype Si atom. They are the same for dextro α-quartz in the *z*(+) setting and for laevo α-quartz in the *z*(−) setting. Because of the twofold site symmetry, β_12_ = β_22_/2 and β_13_ = β_23_/2. To convert to the obverse *r* settings, switch the signs of β_13_ and β_23_ as shown in Table 8 ▸.

**Figure 4:**
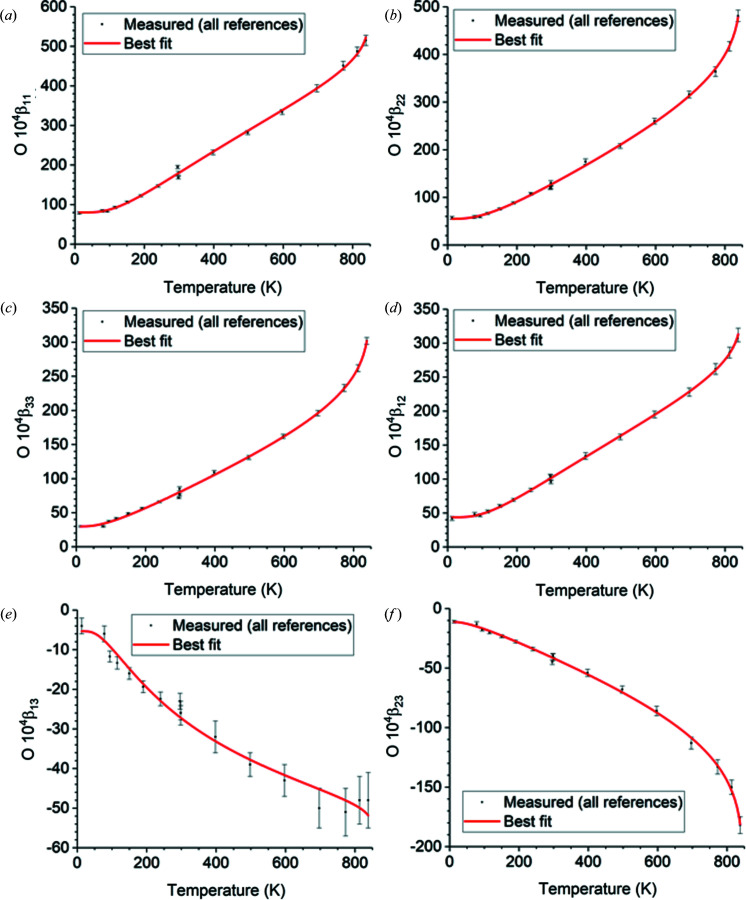
Temperature fits over 13–838 K of the displacement ellipsoid matrix elements of the prototype O atom. They are the same for dextro α-quartz in the *z*(+) setting and for laevo α-quartz in the *z*(−) setting. To convert to the obverse *r* settings, switch the sign of β_13_ and β_23_ as shown in Table 8 ▸.

The temperature fits over 13–838 K of the measured data cited in Table 9 ▸ to equation (10) using the optimal free parameter values in Table 10 ▸ are shown in Fig. 1 ▸ (lattice parameters *a* and *c*), Fig. 2 ▸ (atomic positions), Fig. 3 ▸ (Si displacement ellipsoid) and Fig. 4 ▸ (O displacement ellipsoid). The deviation of the best-fit function from the measured data lies within twice the estimated errors provided by the references and is usually much less.

## 7. Atomic form factors

The non-anomalous X-ray scattering factors *f*_0_ of the Si and O atoms were calculated as a function of *s* = sinθ/λ, where θ is the Bragg angle and λ is the X-ray wavelength, from the five-Gaussian neutral atom fits of Waasmaier & Kirfel (1995 ▸):^2^


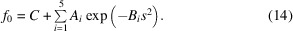


**Figure 5:**
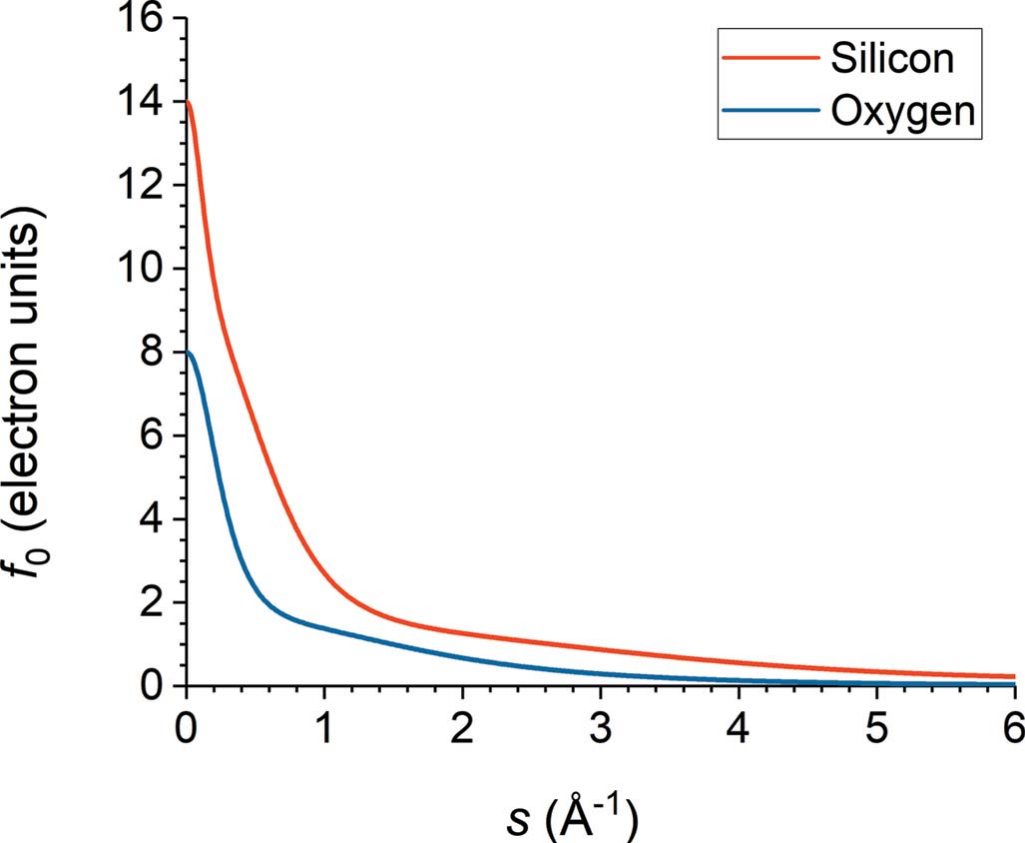
Values of the non-anomalous atomic scattering factor *f*_0_ of Si and O as calculated from the fits of Waasmaier & Kirfel (1995 ▸).

*A*_*i*_, *B*_*i*_ and *C* are the fitting parameters, and *s* is in units of Å^−1^. This fitting function was chosen for high accuracy at large values of *s* up to 6.0 Å^−1^, which are especially important when backscattering Bragg reflections are being considered. Fig. 5 ▸ shows the values of *f*_0_ for Si and O.

The values for anomalous dispersion were calculated using the *abs* program of Brennan & Cowan (1992 ▸), which was originally written in Fortran77 but is now available in a public-domain Python 3 version that was provided by Brennan and is included with our software package. The method published by Cromer & Liberman (1981 ▸) was used to determine the real part *f*′ and the imaginary part *f*′′, which is related to the photoelectric absorption cross section. The *abs* program was also used to determine the contribution to the imaginary part of the scattering factor that arises from Rayleigh and Compton scattering (McMaster *et al.*, 1969 ▸). These are labelled here as *f*_R_ and *f*_C_, respectively. Note that the Compton scattering may not be negligible when X-ray diffraction from light atoms is treated, as is the case in α-quartz. The total atomic scattering factor *f* is therefore

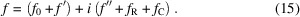




Note that the calculated anomalous dispersion will not be correct near an absorption edge and that the effects of crystal structure on absorption are neglected. This is not a serious problem for α-quartz or sapphire because the absorption edges of silicon, aluminium and oxygen are all far below the energies at which backscattering crystal spectrometers would normally be used. However, for other potentially useful crystals like lithium niobate that contain atoms of higher atomic number, more care might need to be taken if the selected X-ray energy is near an absorption edge of one of the atoms.

## 8. Calculation of the structure factor

In the following, we take the convention that the phase term of a plane wave is exp(−2π*i* **k**·**r**), where **k** is the wavevector and **r** is a position. This convention is used in many standard texts on X-ray diffraction, such as Zachariasen (1945 ▸), Batterman & Cole (1964 ▸) and Authier (2006 ▸). It is also used in standard software packages such as *XOP* (Sánchez del Río & Dejus, 2004 ▸, 2011 ▸). However, other software packages such as the APS *X-ray Server* (Stepanov, 1997 ▸) and *SRW* (Chubar & Elleaume, 1998 ▸) define the phase term of a plane wave as exp(+2π*i* **k**·**r**). Unfortunately, confusion can arise if a structure factor calculated under one of these conventions is input to a program that uses the other.

If the phase term of a plane wave is exp(−2π*i* **k**·**r**) as we assume, the structure factor *F*(*hkl*) for diffraction from the (*hkl*) atomic planes of a crystal is determined by the formula

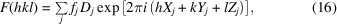

where the index *j* designates a particular atom within the unit cell. *f*_*j*_ is the total atomic scattering factor of the *j*th atom. *D*_*j*_ is the Debye–Waller factor of the *j*th atom as given by equation (2). (*X*_*j*_, *Y*_*j*_, *Z*_*j*_) are the coordinates of the *j*th atom in the lattice coordinate system (**a**, **b**, **c**). The atomic coordinates in the reverse *z* setting are given in Table 4 ▸ for dextro α-quartz and in Table 5 ▸ for laevo α-quartz. See Table 8 ▸ for conversion to the obverse *r* setting.

If the phase term of a plane wave is exp(+2π*i* **k**·**r**), the scattering factors *f*_*j*_ and the exponential terms exp[2π*i*(*hX*_*j*_ + *kY*_*j*_ + *lZ*_*j*_)] are replaced by their complex conjugates.

## 9. Structure of the program

### 9.1. Requirements

The package requires a Python 3 interpreter with the modules __future__, abc, h5py, importlib, math, matplotlib, numpy, os, pandas, pkgutil, re, sys and xlrd.

### 9.2. Code structure and use

#### 9.2.1. Directories

The code is contained within the directory SFC and contains the Python packages general_crystals and Structure_Factor_Calculator. Note that each of these packages contains its own file __init__.py as Python programming rules require.

The directory also contains the following files:

(i) An *Excel* spreadsheet Form_factor_coef f icients.xlsx, which contains the fitting parameters for the atomic scattering factors of Waasmaier & Kirfel (1995 ▸) [see equation (14)].

(ii) Any Python 3 scripts written by the user that apply the modules above.

#### 9.2.2. Definition of the crystal

The crystal is defined in the Python package general_crystals, which contains the module general_crystal.py and all material files. The material files may be named according to each user’s wishes.

For maximum flexibility, an abstract base class called GeneralCrystal is defined in the module general_crystal.py. This includes a basic set of attributes and methods that are useful for any type of crystal:

(i) Crystal system (cubic, tetragonal, orthorhombic, hexagonal, rhombohedral/trigonal, monoclinic, triclinic).

(ii) Unit-cell parameters (sides and angles).

(iii) Determination of lattice vectors (module latt_vec_A).

(iv) Determination of the metric matrix defined in equation (4) (module G_matrix_A2).

(v) Determination of the wavelength, interplanar spacing and Bragg angle for Bragg reflection of X-rays of a given energy from atomic planes of given Miller indices (*hkl*) (module angle_f inder).

GeneralCrystal also includes a set of abstract methods that are named but not implemented:

(i) information: to print out important details about the crystal.

(ii) lattice_unit_cell_params: to set the lattice parameters of the crystal.

(iii) refatom_coordinates: to set the position of the prototype atom in the crystal.

(iv) atoms_init_and_update: to set the element types and determine the positions and thermal vibrations of all the atoms in the crystal.

Instances of an abstract base class cannot be produced, but each material file in the Python package general_crystals defines a subclass of GeneralCrystal from which instances can be created. For example, in the file alphaqua­rtz_dextro_zp.py that defines dextro α-quartz in the *z*(+) setting, we define class AlphaQuartz_Dextro_zp(GeneralCrystal).

In each material file, an implementation for the abstract methods in GeneralCrystal must be provided, but users are free to decide what implementation best suits them. Users may also use the material file to add new model-dependent attributes and methods not provided by GeneralCrystal. Each material file should include the lines

import numpy as np

from .general_crystal import GeneralCrystal

and should also access the modules in Structure_Factor_Calculator as follows:

from Structure_Factor_Calculator.checks import Check

from Structure_Factor_Calculator.atom import Atom

from Structure_Factor_Calculator.dif fraction_environment import Dif f_Environment

from Structure_Factor_Calculator.tools import Tools

The module general_crystal.py also includes a class CrystalFactory. The module __init__.py in the Python package general_crystals imports this class so that all material files in general_crystals can be read. Each module of user-written code in the main directory SFC must include the line

from general_crystals import CrystalFactory

and must also import the definition of the crystal. For example, in our case, the user-written code in SFC contains the line

from general_crystals.alphaqua­rtz_dextro_zp import AlphaQuartz_Dextro_zp

In order to minimize confusion between dextro and laevo α-quartz, we chose to define them in separate material files. Additionally, for quartz of either handedness, we made one material file that includes the full treatment of the displacement ellipsoids, and another material file that treats the thermal vibrations using an isotropic Debye model with a Debye temperature of 470 K for both Si and O atoms. The full list of material files provided for α-quartz is therefore as follows:

(i) alphaqua­rtz_dextro_zp.py: dextro α-quartz in the *z*(+) setting with displacement ellipsoids. Defines class AlphaQuartz_Dextro_zp(GeneralCrystal).

(ii) alphaqua­rtz_dextro_zp_isodwf.py: dextro α-quartz in the *z*(+) setting with the isotropic Debye model. Defines class AlphaQuartz_Dextro_zp_isodwf(GeneralCrystal).

(iii) alphaqua­rtz_laevo_zm.py: laevo α-quartz in the *z*(−) setting with displacement ellipsoids. Defines class AlphaQuartz_Laevo_zm(GeneralCrystal).

(iv) alphaqua­rtz_laevo_zm_isodwf.py: laevo α-quartz in the *z*(−) setting with the isotropic Debye model. Defines class AlphaQuartz_Laevo_zm_isodwf(GeneralCrystal).

Those who prefer the *r* settings will have little difficulty modifying the files above using the conversions in Table 8 ▸.

The class attributes defined in the material files for α-quartz are listed in Table 11 ▸. Other attributes may be added for data checking at the user’s discretion.

Objects of these classes are initialized with the arguments in Table 12 ▸.

When an object of these classes is initialized, the class method set_temp_miller_energy(self, temperature_K, hkl, energy_eV) is called to set the crystal’s temperature and then to determine the unit-cell parameters, the atomic positions, the diffraction parameters, and the atomic species and scattering factors. This method must subsequently be called whenever the crystal’s temperature is changed. When the temperature is unchanged but a different diffracting plane or photon energy is desired, a simpler updating method set_miller_energy(self, hkl, energy_eV) can be called so that temperature-dependent parameters do not need to be recalculated, thus saving time if a large number of Bragg reflections are calculated at the same temperature.

The next set of class methods are specific to our model of α-quartz and may be replaced by more suitable methods for other crystals. The fitting equation (10) is implemented in the class method f itting_equation(self, temp_K, f0, P, Q, n). The symmetry operations of α-quartz are implemented in the class methods screw_matrix(self, atom_coord), two_fold_matrix(self) and three_fold_matrix(self). When displacement ellipsoids are used, the class method beta_matrix_gen(self, element, beta_list = None) organizes the ellipsoid parameters into the full symmetric 3 × 3 matrix for later processing.

The remaining class methods are implementations of the abstract methods of the abstract base class GeneralCrystal. In our material files, these implementations are supported by the set of crystal-specific class methods defined above.

#### 9.2.3. Structure factor code

The structure factor code is contained in the Python package Structure_Factor_Calculator, which consists of the following:

(i) atom.py: defines the class Atom that contains the atomic properties.

(ii) checks.py: defines a simple class Check containing a set of methods for input checking.

(iii) dif fraction_environment.py: defines the class Dif f_Environment that contains the diffraction parameters.

(iv) physical_constants.py: defines a set of physical constants.

(v) structure_factor_calc.py: defines the class Structure_Factor, which contains the methods for calculating the structure factor.

(vi) tools.py: defines a set of miscellaneous methods that are useful for the other files.

(vii) xrpy: the Python package that contains the methods of Brennan & Cowan (1992 ▸) for calculating the anomalous terms of the atomic scattering factors. This includes anomalous dispersion, Compton scattering and Rayleigh scattering.

Especially important are the treatment of the atom, the environment and the structure factor calculation, which are as follows.

#### 9.2.4. Definition of the atom

Each atom is treated as an object of class Atom, which has the attributes in Table 13 ▸. Note that the thermal atomic vibration is treated by inputting either beta_matrix for a displacement ellipsoid or M_and_TD for an isotropic Debye model, but not both.

Objects of class Atom are initialized with the arguments in Table 14 ▸. beta_matrix and M_and_TD are keyword arguments whose default values are None. One but not both of these arguments must be supplied by the user.

An already existing object of class Atom may be updated with the method update, which saves time by skipping the element definition and the reading of the fitting parameters form_factor_coef f icients. Class Atom also includes a method information, which prints out the values of the object’s attributes. data_selecter is the method that reads the values of form_factor_coef f icients from the provided *Excel* spreadsheet Form_factor_coef f icients.xlsx. The method scattering_factor calculates form_factor, f0 and form_factor_fwd for the atom described.

#### 9.2.5. Definition of the environment

The environment includes the parameters that describe the Bragg reflection of X-rays from the crystal at a specified temperature. Class Diff_Environment contains the attributes in Table 15 ▸. Objects of this class are initialized by the arguments in Table 16 ▸.

An existing object of class Dif f_Environment can be updated by calling its method update with the current values of these arguments. Method information prints out the current values of the object’s attributes. Method d_hkl calculates the interplanar spacing for the given Miller indices hkl, and method cell_volume calculates the volume of the unit cell. Note that these methods have been written with all types of crystal structures in mind and not just that of α-quartz.

#### 9.2.6. Structure factor calculation

Class Structure_Factor contains several methods used for determining the structure factor from the description of the crystal, the atoms and the environment:

(i) debye_waller: calculates the Debye–Waller factor of a particular atom in the given crystal for the specified Bragg reflection using displacement ellipsoids.

(ii) isotropic_debye_waller: calculates the Debye–Waller factor of a particular atom in the given crystal for the specified Bragg reflection using an isotropic Debye model.

(iii) atom_scat_phase_DW: multiplies an atom’s atomic scattering factor by the Debye–Waller factor and the phase term in equation (16). This includes a check to avoid un­necessary repetition of calculations for identical atoms.

(iv) F_hkl: calculates the structure factor of the specified Bragg reflection hkl, for the Bragg reflection 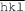 and for forward scattering. Dynamical diffraction calculations of reflectivity generally require all these quantities as inputs, and therefore it is convenient to calculate them all at once.

(v) SF_output: prints out the value of the structure factor along with accompanying information.

## 10. Results

Three examples will show how the structure factor code can provide large numbers of structure factors of α-quartz with minimal effort.

### Comparison of 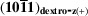 and 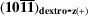


10.1.

A Python 3 script SF-vs-T_Dextro-z+_101.py was saved in the main directory SFC. Its first purpose is to calculate the structure factors of 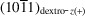 and 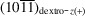 over the entire range of valid temperatures 20–838 K in steps of 1 K. Its second purpose is to compare the structure factors obtained from the full anisotropic displacement ellipsoids with those obtained by assuming an isotropic Debye model of Debye temperature 470 K for both Si and O atoms. An X-ray energy of 10 000 eV was chosen. At each temperature, the script writes the following in a text file:

(i) The Bragg angle θ_B_ = arcsin(λ/2*d*) in degrees (λ is the wavelength of the X-rays and *d* is the spacing of the diffracting atomic planes).

(ii) The squared magnitude and the phase of the structure factor *F*(*hkl*) of the given Bragg reflection.

(iii) The squared magnitude and the phase of the structure factor 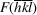 of the negative of the given Bragg reflection.

(iv) The squared magnitude and the phase of the structure factor *F*_0_ for forward scattering.

The script is given in the supporting information.

**Figure 6:**
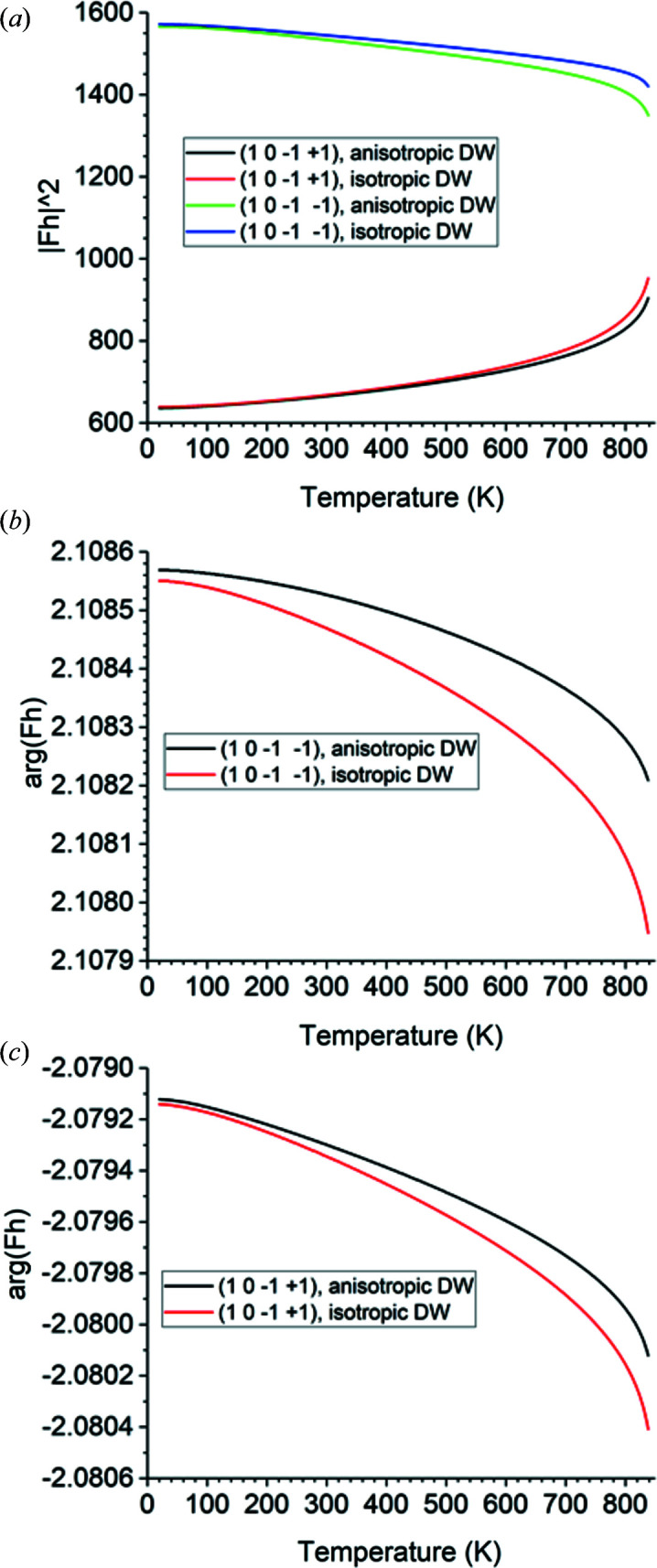
Plots over temperature from 20 to 838 K for α-quartz using both anisotropic displacement ellipsoids (‘anisotropic DW’) and an isotropic Debye model with Debye temperature 470 K for all atoms (‘isotropic DW’). DW stands for Debye–Waller. X-rays of 10 000 eV energy are used. (*a*) The squared magnitude of the structure factors of 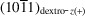 and 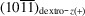 . (*b*) The phase angle in radians of the structure factor of 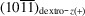 . (*c*) The phase angle in radians of the structure factor of 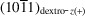 .

The Bragg angle is 10.707° at 20 K and decreases to 10.556° at 838 K. The properties of the structure factors are shown in Fig. 6 ▸. The plot in Fig. 6 ▸(*a*) correctly shows that the 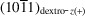 reflection is weaker than the 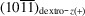 reflection, as pointed out by Glazer (2018 ▸). It also shows, however, that the magnitudes of the two structure factors approach each other as the transition from α-quartz to β-quartz is approached. This is to be expected because, at the transition, the threefold screw axis becomes a sixfold screw axis and these two Bragg reflections therefore become symmetrically equivalent. The isotropic thermal model consistently yields larger squared magnitudes of the structure factors than the anisotropic model, but the two models remain within 1% up to 530 K for 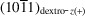 and up to 412 K for 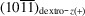 . However, above these temperatures, the isotropic thermal model deviates progressively more from the anisotropic model as the transition is approached. At 838 K, the squared magnitudes of the structure factors calculated by the isotropic model are 5.3% greater than those calculated by the anisotropic model. The phase angles of the structure factors calculated by both thermal models agree to within 0.014%, as shown in Figs. 6 ▸(*b*) and 6 ▸(*c*).

### Comparison of 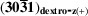 and 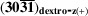


10.2.

A Python 3 script SF-vs-T_Dextro-z+_301.py was saved in the main directory SFC. Its first purpose is to calculate the structure factors of 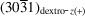 and 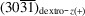 over the entire range of valid temperatures 20–838 K in steps of 1 K. Its second purpose is to compare the structure factors obtained from the full anisotropic displacement ellipsoids with those obtained by assuming an isotropic Debye model of Debye temperature 470 K for both Si and O atoms. An X-ray energy of 10 000 eV was chosen. At each temperature, the script writes the following in a text file:

(i) The Bragg angle in degrees as defined in the previous example.

(ii) The squared magnitude and the phase of the structure factor *F*(*hkl*) of the given Bragg reflection.

(iii) The squared magnitude and the phase of the structure factor 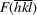 of the negative of the given Bragg reflection.

(iv) The squared magnitude and the phase of the structure factor *F*_0_ for forward scattering.

This script is exactly the same as SF-vs-T_Dextro-z+_101.py except that the Miller indices and the names of the output text files have been updated.

**Figure 7:**
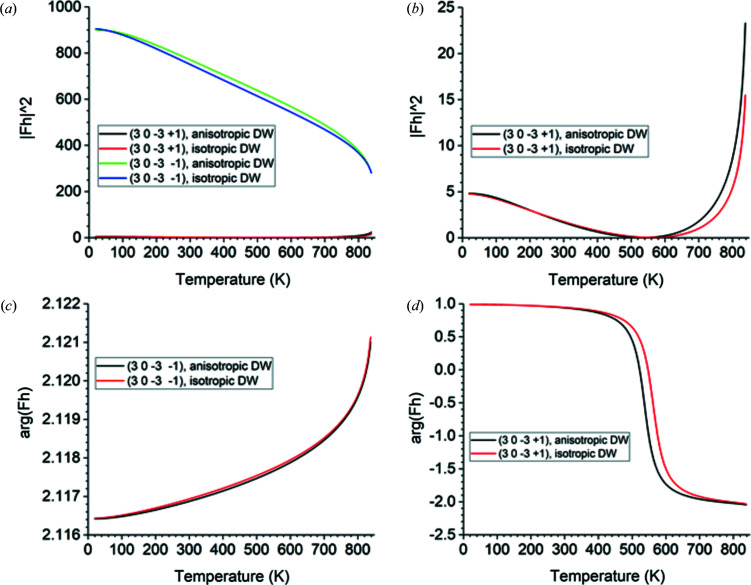
Plots over temperature from 20 to 838 K for α-quartz using both anisotropic displacement ellipsoids (‘anisotropic DW’) and an isotropic Debye model with Debye temperature 470 K for all atoms (‘isotropic DW’). DW stands for Debye–Waller. X-rays of 10 000 eV energy are used. (*a*) The squared magnitude of the structure factors of 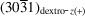 and 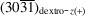 . (*b*) The squared magnitude of the structure factor of 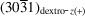 , plotted alone to show where it drops almost to zero and then recovers. (*c*) The phase angle in radians of the structure factor of 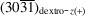 . (*d*) The phase angle in radians of the structure factor of 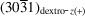 .

The Bragg angle is 26.932° at 20 K and decreases to 26.459° at 838 K. The properties of the structure factors are shown in Fig. 7 ▸. The plot in Fig. 7 ▸(*a*) correctly shows that the 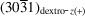 reflection is much weaker than the 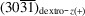 reflection, as pointed out by Glazer (2018 ▸). As in the previous example, these two reflections become symmetrically equivalent in β-quartz; therefore, it is not surprising that the squared magnitudes of their structure factors approach each other just below the transition temperature. With increasing temperature, the squared magnitude of the structure factor of the very weak 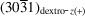 reflection falls almost to zero before re­covering, as seen in Fig. 7 ▸(*b*). This occurs because the total scattering from the three Si atoms very nearly cancels out the total scattering from the six O atoms. At lower temperatures the scattering from the O atoms dominates, while at higher temperatures the scattering from the Si atoms dominates. The minimum value is 0.02533 at 536 K if the anisotropic thermal model is used and 0.02424 at 562 K if the isotropic model is used. In Fig. 7 ▸(*d*), one sees that the minimum in the squared magnitude of this structure factor is accompanied by a change in the phase angle of 3.02 rad, which amounts to almost a complete phase reversal. Finally, Fig. 7 ▸(*c*) shows the phase angle of the stronger reflection 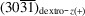 , which is very similar in both the anisotropic model and the isotropic model. The deviation of the isotropic model from the anisotropic model behaves very differently with temperature from that observed in the previous example. For the stronger reflection 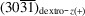 , the isotropic model yields a consistently *weaker* structure factor than the anisotropic model, with the largest deviation being 4.26% at 643 K. The phase angle of the structure factor of this reflection is the same in both thermal models to within 0.006%. For the very weak reflection 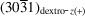 , the disagreement between the two thermal models is very large. This example demonstrates the importance of accurate models of the temperature dependence of a crystal’s atomic positions and thermal vibrations when calculating structure factors of weak reflections.

For three of these four reflections, the isotropic Debye model agrees well with the anisotropic model of thermal vibrations for temperatures below about 450 K, but matches less well as the temperature increases from there to the α → β transition. The only exception is the strong reflection 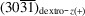 , where the two models agree within less than 5% throughout.

### 10.3. Finding Bragg reflections of α-quartz that backscatter X-rays of a specified energy

This last example, given in the Python script f ind_back_ref_Search.py, shows that backscattering Bragg reflections suitable for X-rays of a specified energy can be found and selected. Dextro α-quartz in the *z*(+) setting at temperatures between 20 and 600 K is assumed. When a backscattering Bragg reflection *hkl* is found, the following parameters are evaluated:

(i) The temperature at which the spacing of the diffracting atomic planes (*hkl*) becomes equal to half the X-ray wavelength.

(ii) The peak reflectivity of the rocking curve in energy.

(iii) The FWHM of the rocking curve in energy.

(iv) The temperature change required to shift the backscattered X-ray energy by 1 meV.

Items (ii) and (iii) are calculated from *F*(*hkl*), 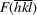 and *F*_0_ using the dynamical diffraction formulas of Batterman & Cole (1964 ▸). Duplicate Bragg reflections related by symmetry are excluded. The results are displayed in Table 17 ▸ for 10 keV X-rays, in Table 18 ▸ for 8.048 keV X-rays (Cu *K*α_1_) and in Table 19 ▸ for 17.479 keV X-rays (Mo *K*α_1_).

## 11. Conclusion

α-Quartz is one of the most promising alternatives to silicon and germanium for the manufacture of X-ray optics. Although α-quartz has a relatively low thermal conductivity compared with silicon, it has already been used successfully in applications where a high heat load is not imposed, particularly as a material for X-ray spectrometers that are operated near backscattering for high energy resolution. Because the trigonal lattice of α-quartz has lower symmetry than the face-centred cubic lattice of silicon, germanium and diamond, α-quartz offers a greater density of backscattering reflections within a given energy range and thus a better match to X-rays of specified energy, such as an emission line. Recent measurements have shown that synthetic α-quartz crystals with a high degree of crystalline perfection can be obtained.

The design of a diffracting crystal X-ray optic requires accurate knowledge of the crystal’s structure factors because these determine its efficiency and bandwidth. For high-resolution X-ray spectrometers, where temperature is used as a tuning parameter, it is important to have a full understanding of its effect on the crystal structure, atomic vibrations and lattice parameters. The temperature dependence of the α-quartz crystal structure is more complex than that of silicon, germanium or diamond because of the anisotropic thermal expansion, the atoms’ movement within the unit cell and their strongly anisotropic thermal vibration. Furthermore, multiple incompatible conventions exist for describing the α-quartz structure.

Building on the careful work of many previous researchers, we have treated the calculation of the structure factors of α-quartz. This treatment includes a full temperature dependence from 20 to 838 K, just short of the 846 K transition to β-quartz. It also accounts for the anisotropy of both the crystal structure and the thermal vibrations of the atoms. For the calculation of large numbers of structure factors, a small set of Python 3 modules has been provided. Python 3 was chosen as the coding language because of its widespread use in X-ray optics software packages and the variety of functionalities offered by its libraries. Different ways of describing and modelling α-quartz can be handled by separate material files, also written in Python 3. Thus, the unresolved question of which convention is best for describing α-quartz is avoided. Tables are provided to convert one convention into another.

Several examples have been provided to illustrate the utility of this code for α-quartz. First, two comparisons of two closely related Bragg reflections, one strong and one weak, as a function of temperature serve to check that the results make sense. In both examples, the full anisotropic treatment of the atomic Debye–Waller factors was compared with an isotropic Debye model. It was shown that the relative discrepancy in the calculated intensity can be significant – several percent if the reflection is strong, and even more if it is weak. The third example was motivated by the intended design of X-ray spectrometers. Here, a desired energy is input, and a list of α-quartz Bragg reflections that reach backscattering at that energy within a given temperature range is printed out, along with their peak reflectivities and FWHM bandpasses. Scripts for the performance of these calculations for backscattering Bragg reflections are shown as models for users and are included in the source distribution (the names of the example scripts and the package containing them may differ slightly from what is described in the paper).

New crystal materials, including the already mentioned sapphire and lithium niobate, can be accommodated with new material files; no change in the basic package is required. Material files for silicon, germanium and diamond are also included in the source distribution. The code can be downloaded from https://github.com/DiamondLightSource/PyCSFex.

## Supplementary Material

An example script. DOI: 10.1107/S1600576722005945/te5094sup1.pdf

## Figures and Tables

**Table 1:** Members of the set of symmetrically equivalent planes {*hkil*} in a hexagonal coordinate system

Miller–Bravais indices	Generated by symmetry operation
(*hkil*)	Identity
(*ihkl*)	120° right-handed rotation about **c**
(*kihl*)	240° right-handed rotation about **c**
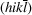	180° rotation about **a**
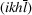	120° right-handed rotation about **c**, then 180° rotation about **a**
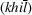	240° right-handed rotation about **c**, then 180° rotation about **a**

**Table 2:** Lattice parameters of α-quartz

Symbol	Meaning	Value at 298 K
*a*	Length of lattice vectors **a** and **b**	4.9137 Å
*c*	Length of lattice vector **c**	5.4047 Å

**Table 3:** Atomic positions in α-quartz D = dextro, *z*(+) setting. L = laevo, *z*(−) setting. The laevo values are equal to one minus the dextro values.

Symbol	Meaning	Value at 298 K (D)	Value at 298 K (L)
*u*	**a** coordinate of prototype Si atom	0.4697	0.5303
*x*	**a** coordinate of prototype O atom	0.4133	0.5867
*y*	**b** coordinate of prototype O atom	0.2672	0.7328
*z*	**c** coordinate of prototype O atom	0.1188	0.8812

**Table 4:** Full list of atomic positions for dextro α-quartz in the z(+) setting Si1 and O1 are, respectively, the prototype Si and O atoms. *u*, *x*, *y* and *z* are given the dextro values in Table 3 ▸.

Atom	Atomic positions	Symmetry operations	Rotation matrix *S*
Si1	(*u*, 0, 0)	Identity	*I*
Si2	(0, *u*, 2/3)	Si1: 1 × left screw about **c**	*M*_3*c*_
Si3	(−*u*, −*u*, 1/3)	Si1: 2 × left screw about **c**	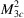
O1	(*x*, *y*, *z*)	Identity	*I*
O2	(−*y*, *x* − *y*, *z* + 2/3)	O1: 1 × left screw about **c**	*M*_3*c*_
O3	(*y* − *x*, −*x*, *z* + 1/3)	O1: 2 × left screw about **c**	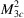
O4	(*x* − *y*, −*y*, −*z*)	O1: 180° rotation about **a**	*M*_2*a*_
O5	(−*x*, *y* − *x*, −*z* + 1/3)	O2: 180° rotation about **a**	*M*_2*a*_ *M*_3*c*_
O6	(*y*, *x*, −*z* + 2/3)	O3: 180° rotation about **a**	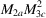

**Table 5:** Full list of atomic positions for laevo α-quartz in the z(−) setting Si1 and O1 are, respectively, the prototype Si and O atoms. *u*, *x*, *y* and *z* are given the laevo values in Table 3 ▸.

Atom	Atomic positions	Symmetry operations	Rotation matrix *S*
Si1	(*u*, 0, 0)	Identity	*I*
Si2	(0, *u*, 1/3)	Si1: 1 × right screw about **c**	*M*_3*c*_
Si3	(−*u*, −*u*, 2/3)	Si1: 2 × right screw about **c**	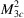
O1	(*x*, *y*, *z*)	Identity	*I*
O2	(−*y*, *x* − *y*, *z* + 1/3)	O1: 1 × right screw about **c**	*M*_3*c*_
O3	(*y* − *x*, −*x*, *z* + 2/3)	O1: 2 × right screw about **c**	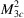
O4	(*x* − *y*, −*y*, −*z*)	O1: 180° rotation about **a**	*M*_2*a*_
O5	(−*x*, *y* − *x*, −*z* + 2/3)	O2: 180° rotation about **a**	*M*_2*a*_ *M*_3*c*_
O6	(*y*, *x*, −*z* + 1/3)	O3: 180° rotation about **a**	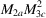

**Table 6:** Elements of the β matrix of the displacement ellipsoid of the prototype Si atom and their values at 298 K as provided by Kihara (1990 ▸) for dextro α-quartz in the *z*(+) setting; also valid for laevo α-quartz in the *z*(−) setting

β_11_	80 × 10^−4^
β_22_	61 × 10^−4^
β_33_	45 × 10^−4^
β_23_	−3 × 10^−4^

**Table 7:** Elements of the β matrix of the displacement ellipsoid of the prototype O atom and their values at 298 K as provided by Kihara (1990 ▸) for dextro α-quartz in the *z*(+) setting; also valid for laevo α-quartz in the *z*(−) setting

β_11_	179 × 10^−4^
β_22_	130 × 10^−4^
β_33_	85 × 10^−4^
β_12_	102 × 10^−4^
β_13_	−26 × 10^−4^
β_23_	−41 × 10^−4^

**Table 8:** Conversion table from the reverse setting to the obverse setting For dextro α-quartz, this converts from the *z*(+) to the *r*(−) setting. For laevo α-quartz, this converts from the *z*(−) to the *r*(+) setting. The displacement ellipsoid matrix β_*ij*_ is converted in the same way for both the Si atoms and the O atoms.

(*hkil*)_obverse_	=	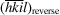
*u*_obverse_	=	−*u*_reverse_
*x*_obverse_	=	−*x*_reverse_
*y*_obverse_	=	−*y*_reverse_
*z*_obverse_	=	*z*_reverse_
(β_11_)_obverse_	=	(β_11_)_reverse_
(β_22_)_obverse_	=	(β_22_)_reverse_
(β_33_)_obverse_	=	(β_33_)_reverse_
(β_12_)_obverse_	=	(β_12_)_reverse_
(β_13_)_obverse_	=	(−β_13_)_reverse_
(β_23_)_obverse_	=	(−β_23_)_reverse_

**Table 9:** Selected references for the temperature dependence of the α-quartz structure

Lattice parameters *a* and *c*, 5–298 K	Integrated from thermal expansion coefficients of Barron *et al.* (1982 ▸), Table 1
Lattice parameters *a* and *c*, 298–838 K	Kihara (1990 ▸), Table 1
Atomic positions and displacement ellipsoids, 94–298 K	Le Page *et al.* (1980 ▸), Table 1
Atomic positions and displacement ellipsoids, 298–838 K	Kihara (1990 ▸), Table 3
Lattice parameters, atomic positions and displacement ellipsoids at 13, 78 and 296 K	Lager *et al.* (1982 ▸), Table I

**Table 10:** Optimal values of the fitting parameters for the lattice parameters, the prototype atomic positions and the prototype displacement ellipsoids of dextro α-quartz in the *z*(+) setting over the temperature range 13–838 K The fits were made to the measured data reported in the publications listed in Table 9 ▸.

Value	*f*_0_	*P*	*Q*	*n*
*a*	4.90137 Å	0.19290 Å	1.80380	0.49873
*c*	5.39806 Å	0.10774 Å	1.72837	0.56369
Si *u*	0.46796	0.14030	3.34189	0.30790
O *t*	−0.05454	2.09511	3.50034	0.28351
Si 10^4^ × β_11_	33.80581	316.85367	1.15728	0.63590
Si 10^4^ × β_22_	35.46433	234.81469	1.24881	0.70758
Si 10^4^ × β_33_	16.72506	166.37647	1.07478	0.61059
Si 10^4^ × β_23_	−1.98183	22.36979	3.52304	0.65136
O 10^4^ × β_11_	80.23419	619.20673	1.00988	0.71444
O 10^4^ × β_22_	55.09116	910.22226	1.66614	0.50506
O 10^4^ × β_33_	29.84124	650.47191	1.75296	0.45406
O 10^4^ × β_12_	43.81216	409.36732	1.13880	0.65112
O 10^4^ × β_13_	−5.35134	−52.36748	0.43392	0.84086
O 10^4^ × β_23_	−11.15409	−718.15015	2.39901	0.33189

**Table 11:** Attributes of Python 3 classes that describe α-quartz

Attribute	Definition
description	Brief definition of the class
TminK	Minimum temperature in kelvin at which the model for the crystal is valid
TmaxK	Maximum temperature in kelvin at which the model for the crystal is valid
temperature_K	Temperature of the crystal in kelvin
crystal_system	Defined here as hexagonal to match the coordinate system
element_list	List showing the element and order of each atom in the crystal’s unit cell
environment	Object of class Dif f_Environment describing diffraction parameters (see text)
atoms	List of objects of class Atom describing each atom in the unit cell (see text)
f it_coef f icients	Dictionary containing the temperature fitting parameters in Table 10 ▸
Tc_K	Temperature of the phase transition from α-quartz to β-quartz
O_Reference_Line_Start	Starting point of the prototype O atom’s position in equation (13)
O_Reference_Line_Vector	Vector in equation (13) along which the prototype O atom moves with temperature
Si_T_Debye_K, O_T_Debye_K (isodwf material files only)	Debye temperatures for vibrations of Si and O atoms, respectively

**Table 12:** Arguments used to initialize Python classes that define α-quartz crystals in the material files

Argument	Definition
temperature_K	Temperature of the crystal in kelvin
hkl	Miller indices of the desired Bragg reflection
energy_eV	Photon energy of the X-rays in electronvolts

**Table 13:** Attributes of class Atom

Attribute	Definition
element	Element of atom (*e.g.* Si or O)
coordinates	Fractional coordinates of atom in unit cell
cartesian_coordinates	Coordinates in a Cartesian system in ångströms
Environment	Object of class Diff_Environment describing diffraction parameters (see text)
form_factor_coef f icients	Fitting parameters for the atomic scattering factor excluding anomalous dispersion, Compton scattering, Rayleigh scattering and Debye–Waller factor; read from the provided *Excel* spreadsheet Form_factor_coef f icients.xlsx
form_factor	Total atomic scattering factor for scattering into the diffracted beam (without Debye–Waller factor)
f0	Atomic scattering factor for scattering into the diffracted beam, excluding anomalous dispersion, Compton scattering, Rayleigh scattering and Debye–Waller factor
form_factor_fwd	Total atomic scattering factor for scattering into the forward direction
beta_matrix	Matrix β of displacement ellipsoid if given
M_and_TD	Atomic mass in atomic mass units and Debye temperature in kelvin if given

**Table 14:** Arguments used to initialize objects of class Atom

Argument	Definition
element	Element of atom (*e.g.* Si or O)
coord	Fractional coordinates of atom in unit cell
environment_obj	Object of class Diff_Environment describing diffraction parameters (see text)
latt_vec_A	Lattice vectors given in Cartesian coordinates in ångströms
beta_matrix	Matrix β of displacement ellipsoid if given
M_and_TD	Atomic mass in atomic mass units and Debye temperature in kelvin if given

**Table 15:** Attributes of class Dif f_Environment

Attribute	Definition
temp_K	Crystal temperature in kelvin
angle_rad	Bragg angle in radians
angle_deg	Bragg angle in degrees
hkl	Miller indices of Bragg reflection
wavelength_A	Wavelength of X-rays in ångströms
d_A	Interplanar spacing of (hkl) atomic planes

**Table 16:** Arguments used to initialize objects of class Dif f_Environment

Argument	Definition
temperature_K	Crystal temperature in kelvin
crystal_system	Type of unit cell (‘Hexagonal’ for α-quartz)
unit_cell_params	Lattice parameters (sides and angles of the unit cell)
angle_plane_wavelength	List containing the Bragg angle in radians, the Miller indices and the X-ray wavelength in ångströms

**Table 17:** Backscattering reflections *hkil* of dextro α-quartz in the *z*(+) setting for 10 keV X-rays *T*_back_ is the temperature at which each set of diffracting atomic planes has a spacing equal to half the X-ray wavelength. *R*_pk_ is the peak reflectivity. FWHM is the full width at half-maximum in terms of energy. mK/meV is the change in temperature in millikelvin required to change the backscattered energy by 1 meV.

*hkil*	*T*_back_ (K)	*R*_pk_	FWHM (meV)	mK/meV
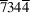	77	0.86	10.88	−24.8
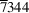	77	0.57	3.07	−24.8
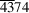	77	0.45	3.07	−24.8
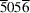	459	0.18	1.40	−7.2

**Table 18:** Backscattering reflections *hkil* of dextro α-quartz in the *z*(+) setting for 8.048 keV X-rays *T*_back_ is the temperature at which each set of diffracting atomic planes has a spacing equal to half the X-ray wavelength. *R*_pk_ is the peak reflectivity. FWHM is the full width at half-maximum in terms of energy. mK/meV is the change in temperature in millikelvin required to change the backscattered energy by 1 meV.

*hkil*	*T*_back_ (K)	*R*_pk_	FWHM (meV)	mK/meV
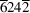	246	0.61	8.48	−9.5
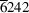	246	0.83	17.96	−9.5
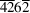	246	0.67	8.48	−9.5
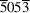	364	0.40	4.34	−8.6

**Table 19:** Backscattering reflections *hkil* of dextro α-quartz in the *z*(+) setting for 17.479 keV X-rays *T*_back_ is the temperature at which each set of diffracting atomic planes has a spacing equal to half the X-ray wavelength. *R*_pk_ is the peak reflectivity. FWHM is the full width at half-maximum in terms of energy. mK/meV is the change in temperature in millikelvin required to change the backscattered energy by 1 meV.

*hkil*	*T*_back_ (K)	*R*_pk_	FWHM (meV)	mK/meV
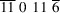	70	0.85	2.29	−15.9
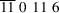	70	0.79	1.64	−15.9
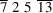	222	0.63	0.89	−6.7
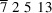	222	0.70	1.08	−6.7
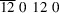	304	0.15	0.26	−3.9
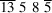	420	0.52	0.63	−3.6
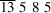	420	0.29	0.36	−3.6
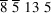	420	0.32	0.36	−3.6
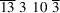	434	0.32	0.38	−3.5
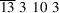	434	0.27	0.34	−3.5
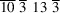	434	0.34	0.38	−3.5
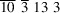	434	0.28	0.34	−3.5
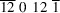	441	0.71	1.16	−3.4

## Footnotes

1 In general, the direction in which a chiral crystal rotates the polarization of light is determined by the handedness of the helix that contains the most strongly polarizable atoms, which may or may not match the handedness of the screw axis. See Glazer & Stadnicka (1986 ▸) for details.

2 We distinguish *s* = sinθ/λ from the also frequently used quantity *q* = 4πsinθ/λ.
