# Supplementary material for: Calculating temperature-dependent X-ray structure factors of α-quartz with an extensible Python 3 package
Source: J Appl Crystallogr. 2022 Jul 28;55(Pt 4):1011–28. doi: 10.1107/S1600576722005945 (PMC9348876; doi:10.1107/S1600576722005945)
Supplement: Supplementary file 1 [file j-55-01011-sup1.pdf]

JOURNAL OF APPLIED CRYSTALLOGRAPHY

Volume 55 (2022)

Supporting information for article:

Calculating temperature-dependent X-ray structure factors of α-quartz with an extensible Python 3 package

John P. Sutter, James Pittard, Jacob Filik and Alfred Q. R. Baron

S1. Example script `SF-vs-T_Dextro-z+_101.py` used in Section 10.1

```
import numpy as np

import sys


from general_crystals import CrystalFactory

from general_crystals.alphaquartz_dextro_zp import AlphaQuartz_Dextro_zp

from general_crystals.alphaquartz_dextro_zp_isodwf import
AlphaQuartz_Dextro_zp_isodwf

from Structure_Factor_Calculator.structure_factor_calc import Structure_Factor


print("sys.path = ",sys.path)

descriptions = CrystalFactory.get_descriptions()


# User parameters


hkl1 = [1,0,1]

hkl2 = [1,0,-1]

tempmin_K = 20

tempmax_K = 838

energy_eV = 10000

OutFile1 = 'SF_Out_hkl1=101.txt'

OutFile2 = 'SF_Out_hkl2=10-1.txt'

OutFileIso1 = 'SFIso_Out_hkl1_101.txt'

OutFileIso2 = 'SFIso_Out_hkl2_10-1.txt'


# Crystal object initialization


AQzD1 = AlphaQuartz_Dextro_zp(tempmin_K,hkl1,energy_eV)

AQzD2 = AlphaQuartz_Dextro_zp(tempmin_K,hkl2,energy_eV)

AQzDIso1 = AlphaQuartz_Dextro_zp_isodwf(tempmin_K,hkl1,energy_eV)

AQzDIso2 = AlphaQuartz_Dextro_zp_isodwf(tempmin_K,hkl2,energy_eV)
```

```
# Calculation and output of structure factors versus temperature

for tempdK in range(tempmin_K,tempmax_K+1):

    AQzD1.set_temp_miller_energy(tempdK,hkl1,energy_eV)
    ThBragg_deg = AQzD1.environment.angle_deg
    SF = Structure_Factor.F_hkl(AQzD1,AQzD1.environment)

    SF_H = SF[0][0]
    SF_H_Mag = np.abs(SF_H)**2
    SF_H_Angrad = np.angle(SF_H)

    SF_Hbar = SF[1][0]
    SF_Hbar_Mag = np.abs(SF_Hbar)**2
    SF_Hbar_Angrad = np.angle(SF_Hbar)

    SF_0 = SF[2][0]
    SF_0_Mag = np.abs(SF_0)**2
    SF_0_Angrad = np.angle(SF_0)

    with open(OutFile1,'a') as out:
        out.write( '{0:d} {1:5f} {2:5f} {3:5f} {4:5f} {5:5f} {6:5f} {7:5f}'.format(
tempdK, ThBragg_deg, SF_H_Mag, SF_H_Angrad, SF_Hbar_Mag, SF_Hbar_Angrad, SF_0_Mag,
SF_0_Angrad ) + '\n' )

    AQzD2.set_temp_miller_energy(tempdK,hkl2,energy_eV)
    ThBragg_deg = AQzD2.environment.angle_deg
    SF = Structure_Factor.F_hkl(AQzD2,AQzD2.environment)

    SF_H = SF[0][0]
    SF_H_Mag = np.abs(SF_H)**2
    SF_H_Angrad = np.angle(SF_H)
```

```
    SF_Hbar = SF[1][0]

    SF_Hbar_Mag = np.abs(SF_Hbar)**2

    SF_Hbar_Angrad = np.angle(SF_Hbar)


    SF_0 = SF[2][0]

    SF_0_Mag = np.abs(SF_0)**2

    SF_0_Angrad = np.angle(SF_0)


    with open(OutFile2,'a') as out:

        out.write( '{0:d} {1:5f} {2:5f} {3:5f} {4:5f} {5:5f} {6:5f} {7:5f}'.format(
tempdK, ThBragg_deg, SF_H_Mag, SF_H_Angrad, SF_Hbar_Mag, SF_Hbar_Angrad, SF_0_Mag,
SF_0_Angrad ) + '\n' )


    AQzDIso1.set_temp_miller_energy(tempdK,hkl1,energy_eV)

    ThBragg_deg = AQzDIso1.environment.angle_deg

    SF = Structure_Factor.F_hkl(AQzDIso1,AQzDIso1.environment)


    SF_H = SF[0][0]

    SF_H_Mag = np.abs(SF_H)**2

    SF_H_Angrad = np.angle(SF_H)


    SF_Hbar = SF[1][0]

    SF_Hbar_Mag = np.abs(SF_Hbar)**2

    SF_Hbar_Angrad = np.angle(SF_Hbar)


    SF_0 = SF[2][0]

    SF_0_Mag = np.abs(SF_0)**2

    SF_0_Angrad = np.angle(SF_0)


    with open(OutFileIso1,'a') as out:

        out.write( '{0:d} {1:5f} {2:5f} {3:5f} {4:5f} {5:5f} {6:5f} {7:5f}'.format(
tempdK, ThBragg_deg, SF_H_Mag, SF_H_Angrad, SF_Hbar_Mag, SF_Hbar_Angrad, SF_0_Mag,
SF_0_Angrad ) + '\n' )
```

```
    AQzDIso2.set_temp_miller_energy(tempdK,hkl2,energy_eV)

    ThBragg_deg = AQzDIso2.environment.angle_deg

    SF = Structure_Factor.F_hkl(AQzDIso2,AQzDIso2.environment)


    SF_H = SF[0][0]

    SF_H_Mag = np.abs(SF_H)**2

    SF_H_Angrad = np.angle(SF_H)


    SF_Hbar = SF[1][0]

    SF_Hbar_Mag = np.abs(SF_Hbar)**2

    SF_Hbar_Angrad = np.angle(SF_Hbar)


    SF_0 = SF[2][0]

    SF_0_Mag = np.abs(SF_0)**2

    SF_0_Angrad = np.angle(SF_0)


    with open(OutFileIso2,'a') as out:

        out.write( '{0:d} {1:5f} {2:5f} {3:5f} {4:5f} {5:5f} {6:5f} {7:5f}'.format(
tempdK, ThBragg_deg, SF_H_Mag, SF_H_Angrad, SF_Hbar_Mag, SF_Hbar_Angrad, SF_0_Mag,
SF_0_Angrad ) + '\n' )
```
